# The mediating effect of sleep duration on metabolic syndrome severity in adults: a structural equation modeling approach

**DOI:** 10.1186/s12902-024-01611-7

**Published:** 2024-05-29

**Authors:** Niloufar Hemati, Shabnam Satari, Habibolah Khazaie, Yahya Salimi, Farid Najafi, Yahya Pasdar, Mitra Darbandi

**Affiliations:** 1 Internal Medicine Department, Kermanshah University of Medical Sciences, Kermanshah, Iran; 2 Kermanshah University of Medical Sciences, Kermanshah, Iran; 3 Sleep Disorders Research Center, Kermanshah University of Medical sciences, Kermanshah, Iran; 4 Social Development and Health Promotion Research Center, Health Institute, Kermanshah University of Medical Sciences, Kermanshah, Iran; 5 Research Center for Environmental Determinants of Health (RCEDH), Health Institute, Kermanshah University of Medical Sciences, Kermanshah, Iran; 6 Student Research Committee, Kermanshah University of Medical Sciences, Kermanshah, Iran

**Keywords:** Sleep duration, Metabolic syndrome severity, Structural equation modeling

## Abstract

**Background:**

Metabolic syndrome (MetS) is associated to sleep duration. It is crucial to identify factors that disrupt sleep regulation. The study aimed to assess the indirect effect of risk factors related to MetS severity through sleep duration by utilizing a structural equation model (SEM).

**Methods:**

The study involving 3,935 adults from the baseline data of the Ravansar Non-Communicable Disease (RaNCD) cohort study. MetS severity scores were the outcome variables. SEM was employed to explore the relationships, utilizing IBM SPSS and AMOS version 23.

**Results:**

The mean MetS severity score was higher in women compared to men (0.25 vs. 0.16, *P* = 0.003). In men, socioeconomic status (SES) has a positive direct effect (β = 0.048) and a negative indirect effect (β=-0.006) on MetS severity. Increased physical activity is directly (β=-0.036) and indirectly (β=-0.093) associated with reducing MetS severity. Nap duration is directly linked to an increase (β = 0.072) but has an indirect effect (β=-0.008) in decreasing MetS severity. In women, SES has a direct (β=-0.020) and indirect (β=-0.001) inverse relationship with MetS severity. Increased physical activity is directly (β=-0.048) and indirectly (β=-0.036) associated with decreasing MetS severity in women. Nap duration is directly associated with an increase in MetS severity (β=-0.018) but indirectly contributes to its reduction (β=-0.002). Sleep duration not only directly affects MetS severity but is also influenced by age, SES, physical activity, obesity and nap duration.

**Conclusion:**

Physical activity, SES, and nap duration directly and indirectly effect the MetS severity. Sleep duration was recognized as a mediating variable that supports the indirect effects.

## Introduction

The optimal sleep duration for adults is 7–8 h according to the recommendation of the National Sleep Foundation [1]. Sleep plays a crucial role in maintaining body homeostasis and regulating physiological, hormonal, and psychological processes [2, 3]. Disruption in the quantity and quality of sleep may lead to hormonal changes and contribute to adverse health outcomes such as hypertension, type 2 diabetes mellitus (T2DM), cancer, and mortality [4–7]. Additionally, sleep duration has been linked to metabolic syndrome (MetS), a cluster of metabolic disorders including obesity, hypertension, hypertriglyceridemia, low and high-density lipoprotein (LDL and HDL) cholesterol, and hyperglycemia [8, 9].

Several studies have investigated the direct association between sleep duration and MetS, with sleep potentially acting as a mediating factor. Factors such as age, shift work, socio-economic status (SES), obesity, and daily physical activity level can influence the duration of sleep [10–13]. For instance, a cross-sectional study on Malaysian manufacturing workers aged 40–65 years revealed a higher prevalence of MetS among night shift workers, with sleep quality identified as a mediating factor in this relationship [10]. Additionally, research utilizing structural equation model (SEM) analysis has indicated that sleep duration serves as a significant mediating factor in the development of overweight and obesity [13, 14]. A study involving 380,055 participants in the UK demonstrated that poor sleep, when combined with low physical activity, exacerbates the risks of all-cause and cause-specific mortality, suggesting potential synergistic effects [15]. On the contrary, research has shown a direct relationship between long sleep duration, good sleep quality, and physical activity [16]. Furthermore, lower SES has been linked to shorter sleep duration, longer sleep latency, increased sleep fragmentation, and greater variability in sleep onset and latency. Conversely, higher income, economic well-being, and education levels are correlated with enhanced sleep efficiency and longer sleep duration [17].

In general, while sleep itself is a significant factor influencing MetS, it can also be disrupted by various other factors. Identifying these factors that impact sleep regulation is crucial for improving sleep habits. Therefore, the study aimed to assess the indirect effect of risk factors related to MetS severity through sleep duration by utilizing a SEM approach.

## Methods

### Study design and participant

This is a cross-sectional study based on baseline data from the Ravansar Non-Communicable Disease (RaNCD) cohort study conducted in western Iran. The RaNCD study is a component of the Prospective Epidemiological Research Studies in Iran (PERSIAN). For more information, you can visit https://persiancohort.com/. The initial phase of data collection took place in 2014, and the study included 10,047 adults aged between 35 and 65 years who were permanent residents of Ravansar. The cohort profile study, which was published in 2019, provides a detailed overview of the design of the RaNCD study [18].

In this study, all participants from the baseline phase of the RaNCD study were initially included (*n* = 10,047). However, participants with specific conditions such as cardiovascular diseases (CVDs) (*n* = 1,709), gestational diabetes (*n* = 194), thyroid disorders (*n* = 560), cancer (*n* = 58), depression (*n* = 125), incomplete information (*n* = 1,574), inadequate energy intake (< 500 or ≥ 4200 kcal/day) (*n* = 800), alcohol consumption (*n* = 328), current smokers (*n* = 670), and pregnant women (*n* = 94) were subsequently excluded. As a result, the final sample size for this study was 3,935.

### Data collection

Socio-demographic characteristics were gathered through digital questionnaires and completed by skilled interviewers. SES was determined using 18 items, which included factors like education level, type of residence, housing conditions, and wealth-related assets. The SES calculation was conducted using the principal component analysis (PCA) method. Subsequently, participants were categorized into three groups ranging from the lowest to the highest SES level [19].

Physical activity levels were assessed utilizing the Persian Cohort Standard Questionnaire and expressed in terms of MET/hours per day. The 24-hour physical activity assessment included sports, work, and leisure activities on a typical weekday, and was divided into three groups based on METs: low (24-36.5), moderate (36.6–44.9), and vigorous (≥ 45) [20].

Measurements such as body mass index (BMI), Body Fat Mass (BFM), and waist circumference (WC) were acquired using a Bio-Impedance Analyzer BIA (Inbody 770, Inbody Co, Seoul, Korea). Biochemical data, including triglyceride (TG), Total cholesterol (TC), HDL, LDL, and fasting blood sugar (FBS), were assessed following a 12-hour fast. The systolic and diastolic blood pressure (SBP and DBP) of the participants were taken while seated on a chair using the standard method after a 10-minute rest, measuring both the right and left arm [18].

### Habitual sleep parameters

Data on self-reported habitual sleep parameters was collected using the Pittsburgh Sleep Quality Index (PSQI-P) questionnaire, which includes inquiries about various sleep-related aspects such as night sleep hours, sleep latency, morning wake-up time, daytime naps, night shifts, leg restlessness, use of sleeping pills, and instances of dozing off [21]. Daytime nap was defined as taking daily naps regularly, with regularity defined as equal to or greater than three times per week. Night shift was characterized as working for at least 6 h between 9 PM and 6 AM. Leg restlessness referred to experiencing restlessness in the legs while asleep. Regular use of sleeping pills was defined as more than twice a week. Sleep duration was quantitatively measured over a 24-hour period.

### Metabolic syndrome severity score

The MetS severity score was computed by assigning weights to the MetS components (HDL, LDL, WC, TG, Glucose, and SBP) based on the formula outlined by DeBoer et al., as shown below [22]:

Men = − 5.5459 + 0.0135× WC – 0.0278 × HDL + 0.0054 × SBP + 0.8340 × ln (TG) + 0.0105×Glucose.

Women = − 7.7516 + 0.0162×WC − 0.0157× HDL + 0.0084× SBP + 0.8872 × ln (TG) + 0.0206×Glucose.

### Statistical analysis

The information of participants included reporting the mean ± standard deviation for continuous variables and the frequency (%) for categorical variables. Normality of the data was assessed in AMOS software using skewness and kurtosis. It is important to note that variables with skewness between + 3 and − 3, and kurtosis between + 10 and − 10 were considered normal, and all variables utilized in the model were found to be normal.

Structural Equation Modeling (SEM) is a key method for analyzing complex data and examining direct and indirect effects of a set of variables on outcomes. It allows for the analysis of various variables that demonstrate the simultaneous effects of variables within a theory-based framework. SEM enables the testing of theoretical models’ validity within specific populations using data.

**Fig. 1:**
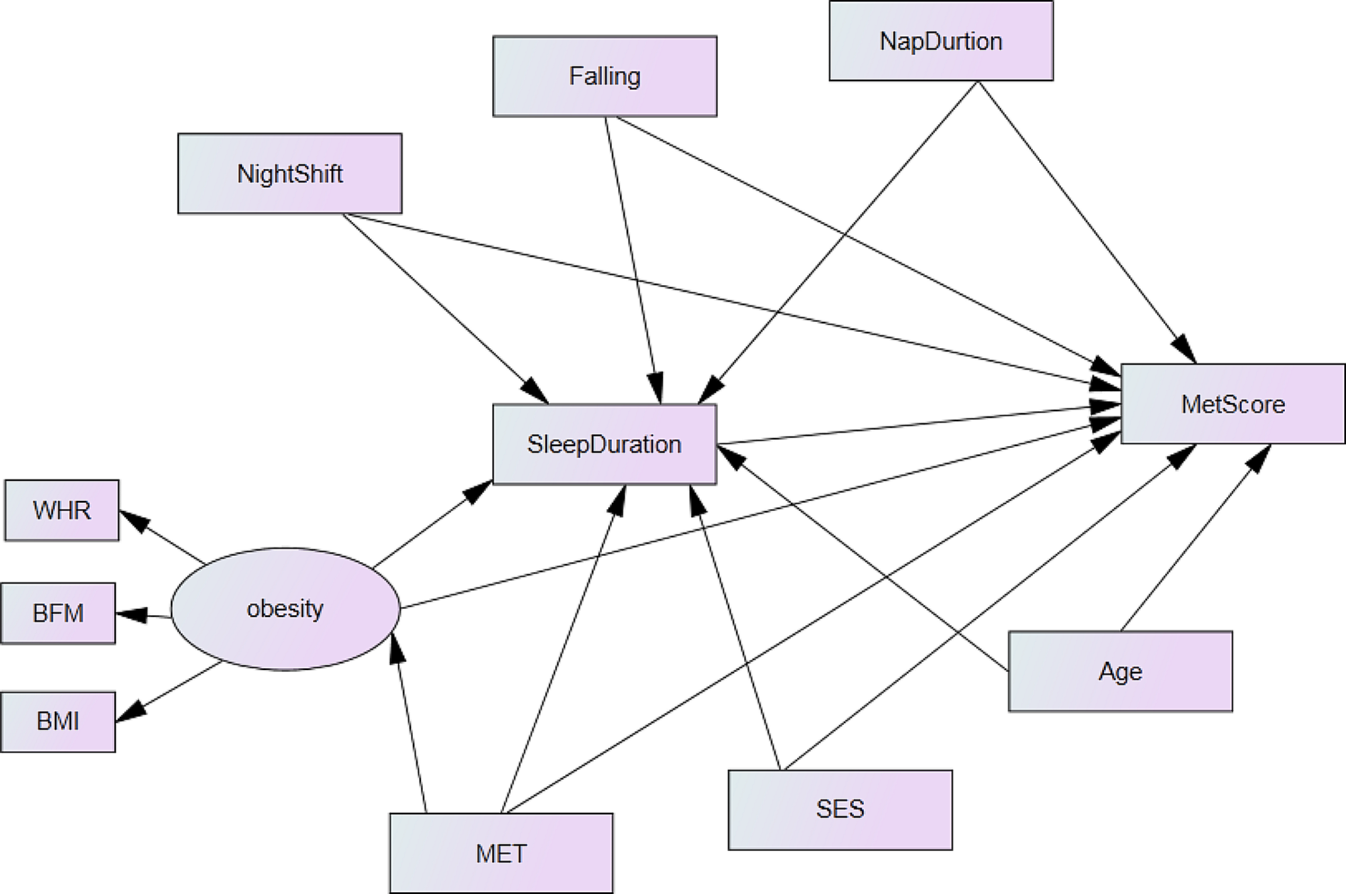
A conceptual model for the association of metabolic syndrome severity score and independent variables. BMI: body mass index; WC: waist circumference; BFM: body fat mass; MET: physical activity; SES: socioeconomic status; Falling: Sleep latency; Nap duration: The duration of nap

The study’s conceptual model, as depicted in Fig. 1, includes a latent variable named obesity composed of three indicator variables (BMI, WC, and BFM). Other variables in the model were observed variables such as age, SES, physical activity, night shift, falling incidents, nap duration, sleep duration, and MetS score. The MetS score served as the dependent variable and primary outcome of the study. Due to significant differences in MetS scores between women and men in the study, separate models were presented for each gender.

To assess the model fit, indices such as the comparative fit index (CFI), incremental fit index (IFI), normed fit index (NFI) at or above 0.90, and root mean square error of approximation (RMSEA) at or below 0.08 were utilized. Model estimates were generated using maximum likelihood estimation (MLE). In all analyses, significance was set at *P* < 0.05. Data management and statistical analyses were conducted using IBM SPSS and AMOS version 23.

## Results

A total of 3,935 participants who met the inclusion criteria for this study were analyzed. Table 1 shows the basic characteristics among men and women. The mean age of the participants was 46.63 ± 8.10 years, and there was no statistically significant difference between men and women (*P* = 0.451). Overall, 2,125 (54.0%) participants were men, and 1,810 (46.0%) were women. Additionally, 2,661 (67.6%) of the participants were rural, and 1,274 (32.3%) were urban. The level of physical activity was significantly different between men and women, with high physical activity being more prevalent in men than in women (37.4% vs. 26.8%, *P* < 0.001).

**Table 1:** Characteristics of study participants

Variables	Total (*n* = 3,935)	Men (*n* = 2,125)	Women (*n* = 1,810)	*P* value*
	Mean ± S.D or Frequency (%)			
Age (year)	46.63 ± 8.10	46.62 ± 8.01	46.64 ± 8.17	0.451
Place of residence				
Rural	2661 (67.62)	1404 (66.07)	1257 (69.45)	0.024
Urban	1274 (32.38)	721 (33.93)	553 (30.55)	
Socioeconomic status				
Low	658 (16.72)	222 (10.45)	436 (24.09)	< 0.001
Moderate	1635 (41.55)	757 (35.62)	878 (48.51)	
High	1642 (41.73)	1146 (53.93)	496 (27.40)	
Physical Activity (met/hour per day)				
Light	1281 (32.55)	796 (37.46)	485 (26.80)	< 0.001
Moderate	1824 (46.35)	642 (30.21)	1182 (65.30)	
High	830 (21.10)	687 (32.33)	143 (7.90)	
Metabolic syndrome	1022 (25.97)	422 (19.86)	600 (33.15)	< 0.001
Metabolic syndrome severity score	0.21 ± 0.88	0.25 ± 0.80	0.16 ± 0.9	0.003
Body Mass Index (kg/m^2^)	27.51 ± 4.49	26.43 ± 3.98	28.78 ± 4.72	< 0.001
Waist Circumference (cm)	97.21 ± 10.26	96.15 ± 9.60	98.43 ± 10.85	< 0.001
Body Fat Mass (kg)	24.96 ± 9.31	21.53 ± 8.11	28.99 ± 9.10	< 0.001
Sleep duration (h/day)	7.06 ± 1.19	6.98 ± 1.19	7.14 ± 1.20	< 0.001
Sleep latency *(*Min*)*	34.50 ± 31.10	27.71 ± 24.40	41.89 ± 34.77	< 0.001
Nap duration *(*Min*)*	65.83 ± 46.45	66.48 ± 48.24	65.55 ± 44.21	0.740
Night shift work *(*Min*)*	34.14 ± 6.51	34.55 ± 10.21	32.4 ± 11.41	0.859
Leg Restlessness	218 (5.54)	96 (4.52)	122 (6.74)	0.001
Dozing off during the day	1306 (33.19)	741 (34.87)	565 (31.22)	0.015
Use sleeping pills	86 (2.19)	40 (1.88)	46 (2.54)	0.159
Morning wakeup (hour)	6.72 ± 1.21	6.52 ± 1.19	6.94 ± 1.20	< 0.001
Night sleep (hour)	9.44 ± 10.90	10.63 ± 10.05	8.04 ± 8.02	< 0.001

*P- value was obtained t-test and Chi – square test

The prevalence of MetS in women was significantly higher than in men (33.15% vs. 19.86%; *P* < 0.001). The severity of MetS was significantly higher in women than in men (*P* = 0.003). Moreover, the average sleep duration was significantly longer in women than in men (*P* < 0.001), and sleep latency was also significantly higher in women than in men (27.71 vs. 41.89 min; *P* < 0.001).

**Fig. 2:**
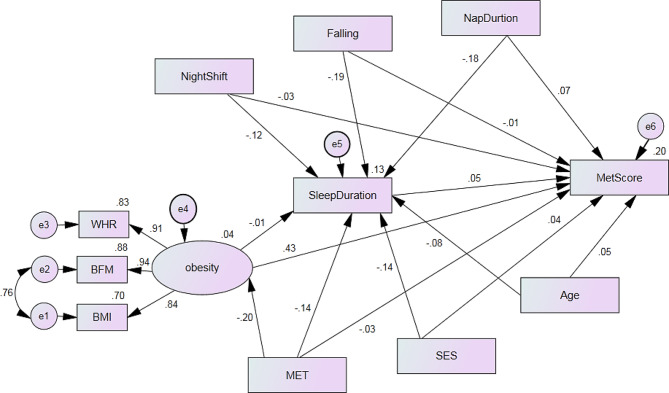
Association of metabolic syndrome severity score and independent variables measured by the structural equation model in men. (Model Fit: RMSEA = 0.841, NFI = 0.910, IFI = 0.902, CFI = 0.902). BMI: body mass index; WC: waist circumference; BFM: body fat mass; MET: physical activity; SES: socioeconomic status; Falling: Sleep latency; Nap duration: The duration of nap

**Fig. 3:**
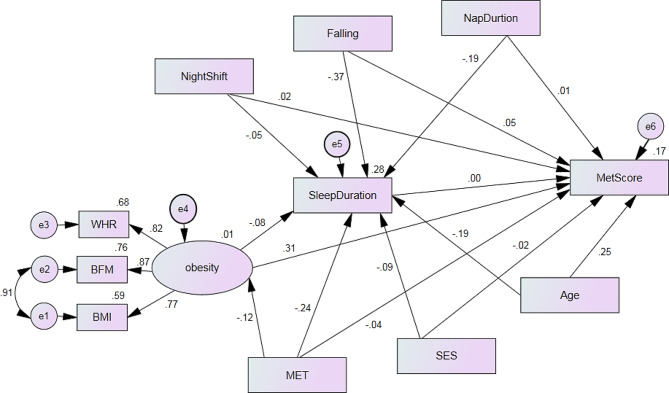
Association of metabolic syndrome severity score and independent variables measured by the structural equation model in women. (Model Fit: RMSEA = 0.076, NFI = 0.949, IFI = 0.953, CFI = 0.953). BMI: body mass index; WC: waist circumference; BFM: body fat mass; MET: physical activity; SES: socioeconomic status; Falling: Sleep latency; Nap duration: The duration of nap

The variables used in the model for men and women were identical. The fit indices of the model were acceptable, confirming its validity. The R^2^ value for the main dependent variable (MetS severity score) was 0.23 in men, indicating that the variables in the model explain 23% of the variance in the outcome (Fig. 2). Similarly, the R^2^ value for the main dependent variable (MetS severity score) was 0.20 in women (Fig. 3).

Direct and indirect associations and the role of the mediation variable (sleep duration) in models related to women and men are presented in Table 2. Increasing age was directly associated with an increase in MetS severity (β_for men_ = 0.059, β_for women_ = 0.259, *P* < 0.05) and indirectly associated with a decrease in MetS severity in both men and women (β_for men_ = -0.004, β_for women_= -0.010, *P* < 0.05). Lower socioeconomic status (SES) was directly and indirectly associated with an increase in MetS severity in women, while in men, this relationship only indirectly increased MetS severity.

**Table 2:** Association of metabolic syndrome severity score and independent variables measured by the structural models model

MetS severity score	Estimate Standardized Coefficient					
	Men			Women		
	Direct effect	Indirect effect	Total effect	Direct effect	Indirect effect	Total effect
Age	0.059 *	-0.004*	-0.051*	0.259 *	-0.010	0.249 *
SES	0.048	-0.006	0.032	-0.020	-0.001	-0.023
Physical Activity	-0.036 *	-0.093*	-0.126 *	-0.048 *	-0.036 *	-0.078 *
Obesity	0.439 *	-0.001	0.430 *	0.315 *	-0.001 *	0.315*
Sleep latency *(*Min*)*	-0.001	-0.009	-0.017	0.050*	-0.004	0.053 *
Nap duration *(*Min*)*	0.072 *	-0.008*	0.061 *	0.018	-0.002	0.011
Night shift work *(*Min*)*	-0.031	-0.005	-0.033	0.020	0.001	0.018
Sleep Duration	0.050 *	--	0.045 *	0.010	--	0.010

* P. value < 0.05

The direct effect of physical activity on MetS severity was − 0.036, the indirect effect was − 0.093, and the total effect was − 0.126. Consequently, increasing physical activity was directly and indirectly associated with decreasing MetS severity in men, with similar associations observed in women. The direct effect of obesity on MetS severity was 0.459, the indirect effect was − 0.001, and the total effect was 0.430 in men. Similarly, in women, obesity was directly associated with an increase in MetS severity and indirectly associated with a decrease in MetS severity.

The direct and indirect effects of night shift work on MetS severity were reversed and not statistically significant. Nap duration increased MetS severity in a direct effect (β_direct_ = 0.072). However, after including sleep duration as a mediating factor between nap duration and MetS severity, the MetS severity decreased (β _indirect_= -0.008) in men. Overall, it was observed in the total effect that daytime nap duration was associated with MetS severity (β_total_ = 0.061) in men, with similar associations observed in women.

An increase in sleep duration was directly associated with an increase in the MetS severity score in both men and women, with this increase being significant in men. In both models, it was observed that sleep duration was influenced by the variables of age, SES, physical activity, obesity, night shift work, sleep latency, and nap duration, and it affected MetS severity as a mediating variable.

## Discussion

Our results indicate that the established risk factors associated to the MetS severity have not only a direct effect but also an indirect effect through a mediating variable that can influence the outcome. In the present study, sleep duration was identified as a mediating variable. The sleep duration plays a mediating role between the MetS severity and the studied risk factors, including age, SES, physical activity, obesity, night shift work, and duration of naps.

In this study, an increase in physical activity was found to be associated with a reduction in the MetS severity in both men and women, both directly and indirectly through its impact on sleep duration. Various studies have demonstrated the relationship between physical activity, sleep duration, MetS severity, weight, and obstructive sleep apnea (OSA) [11, 16, 23]. Additionally, the research by Huang et al. demonstrates that the negative connections between inadequate sleep and risks of all-cause and cause-specific mortality are worsened by low physical activity, indicating potential synergistic effects (24). By enhancing physical activity, it is possible to regulate sleep duration and consequently alleviate MetS severity. This highlights the significance of exploring the indirect connection and the role of the mediating variable.

The study’s results revealed that lower SES was directly and indirectly linked to an escalation in MetS severity among women, while in men, this relationship only indirectly intensified the MetS severity. A study on a working population in Spain indicated that low SES influenced the prevalence of metabolic syndrome in both genders, with a greater impact on women than men [25]. Among rural Chinese adults, a notable prevalence of MetS was observed among older individuals, those with lower income levels, lower educational attainment, or unemployment [26]. Notably, lower SES was associated with short sleep duration, long sleep duration, and napping [12]. A cross-sectional study indicated that individuals with lower income, lower educational levels, and severe food insecurity reported significantly shorter sleep durations [27]. These results indicate that sleep is significantly influenced by the SES. The SES, apart from its direct impact on the metabolic condition, indirectly contributes to an escalation in the MetS severity through its effect on sleep.

The study revealed that the effect of night shift work, both directly and indirectly, had an inverse correlation with the MetS severity in men. Conversely, in women, MetS severity increased directly and indirectly with the rise in night shift work, although this increase was not statistically significant. Findings from Korsiak et al.‘s study in Canada indicated that sleep duration served as a crucial intermediary factor between work shifts and MetS, where the indirect effect of shift work on MetS outweighed the direct effect. In the direct effect, shift work raised the likelihood of developing MetS by 1.18 times (OR_direct_= 1.18). Upon introducing sleep duration as a mediating factor between work shifts and MetS into the model, the odds of developing MetS further increased (OR_indirect_= 2.25). Moreover, the total effect analysis demonstrated a strong association between shift work and MetS (OR _total_= 2.72) [28]. A study involving Malaysian manufacturing workers identified night shift work as a significant risk factor for MetS [10]. Research on Taiwanese hospital employees and railway workers in southwest China indicated that long-term shift work, particularly, heightened the risk of developing MetS [29, 30]. A meta-analysis conducted in 2021 also indicated a positive correlation between shift work and the risk of MetS, which remained significant regardless of adjusting for sleep duration [31]. Discrepancies in findings could be attributed to variations in the study populations. The studies mentioned focused on factory workers and hospital staff with prolonged exposure to regular work shifts over many years. In this study, some participants had intermittent night shifts rather than continuous ones, potentially contributing to the differing results compared to previous research. Longitudinal studies are required to further investigate the relationship between night shifts and the syndrome.

Based on our findings, daytime nap duration exhibited a direct effect of increasing MetS severity (β_direct_ = 0.072). However, upon introducing sleep duration as a mediating factor between nap duration and MetS severity, there was a decrease in MetS severity (β_indirect_= -0.011). Overall, the total effect analysis revealed an association between daytime nap duration and MetS severity (β_total_ = 0.061) in men, with similar associations observed in women. A dose-response meta-analysis conducted in 2016 indicated a J-curved relationship between napping duration and the risk of MetS, suggesting that longer naps are linked to an increased risk of MetS [32]. He et al. also noted that extended daytime naps are associated with a heightened risk of MetS, while short naps have no impact on MetS [33]. Given the lack of research evaluating the indirect effects of napping on MetS, it is imperative to explore these associations in future studies.

In the study involving women, there was a significant increase in the MetS severity with longer sleep onset latency, both directly and overall. Notably, the time taken to fall asleep was significantly longer in women compared to men (27.71 vs. 41.89 min; *P* < 0.001). Age is a well-known uncontrollable factor that can impact this, as the average time taken to fall asleep tends to increase with age, making older individuals more prone to sleep issues [34]. Various studies have shown that smoking can also prolong sleep onset in both men and women. Sahlin et al. found that women with alcohol dependency experienced longer sleep latency [35]. Other factors such as night-shift work, daytime sleepiness, sleep disorders like sleep apnea or restless legs syndrome, and periodic movement disorders were considered and eliminated as causes. Additionally, prolonged sleep latency, which leads to reduced sleep duration, can be linked to vitamin D deficiency and chronic insomnia [36, 37]. Moreover, a study conducted by Zhong et al. in 2022 reveals that prolonged sleep latency is linked to a higher risk of high blood pressure in both men and women [38]. Addressing this issue involves identifying and treating chronic insomnia, maintaining a balanced diet rich in essential nutrients, and addressing factors contributing to delayed sleep onset.

A meta-analysis study highlighted a significant association between short sleep duration and obesity, whereas long sleep duration showed no impact on obesity occurrence in adults [39]. Conversely, obesity influences sleep duration and quality due to respiratory conditions [40, 41]. Thus, obesity not only directly contributes to the onset of MetS but also indirectly impacts its development or exacerbates its severity through its influence on sleep duration.

Previous studies have indicated a robust U-shaped relationship between sleep duration and the MetS severity [8, 42]. Prolonged sleep duration is significantly associated with an increased risk of heart disease, stroke, and overall cardiovascular disease (CVD) and MetS [43–45]. However, previous studies primarily focused on the direct effects of sleep duration on MetS and its severity. Various mechanisms are suggested to explain the association between sleep duration and MetS. Various pathways connect sleep duration to MetS. Inadequate sleep could result in the endocrine modifications outlined below by impacting carbohydrate metabolism, the hypothalamo-pituitary-adrenal axis, and sympathetic activity. Reduced glucose tolerance and insulin sensitivity could elevate glucose levels; heightened ghrelin levels, reduced leptin levels, and increased appetite are linked to larger WC; while elevated cortisol levels are connected to higher blood pressure [46, 47]. Individuals who sleep for a short duration often show increased levels of high-sensitivity C-reactive protein and Interleukin-6, which are associated with cardiovascular events [48, 49]. A prolonged duration of sleep is associated to sleep disruption, which can result in multiple health outcomes, such as metabolic changes [50]. People who sleep for an extended period also have limited time for exercise, which may contribute to this link [51]. Short and long sleep durations both demonstrate reciprocal relationships with circadian rhythm, a risk factor for metabolic disorders [52, 53]. Nevertheless, it has been recommended to validate the mechanism of a study through Mendelian randomization design, utilizing measured genetic variations to establish the causality of the relationship.

The cross-sectional design of the current study poses a limitation as it precludes the exploration of causal relationships. Furthermore, variations in sleep duration due to seasonal changes and potential recall bias may introduce information bias. It’s important to note that this survey was conducted in a small city and may not be generalized to the entire population of Iran. Moreover, genetic factors were not controlled for in the current study. Future studies could explore other risk factors for MetS and sleep duration, including genetic factors, utilizing a SEM approach. Nonetheless, the strengths of our study include a large sample size and the inclusion of urban and rural populations. This study represents the first SEM analysis conducted on the Kurdish population in Iran, serving as a valuable reference for future research and facilitating ethnic comparisons.

## Conclusion

The study findings indicate that in men, SES has a positive direct effect and a negative indirect effect on the MetS severity. Increased physical activity is linked directly and indirectly to reduced MetS severity. Nap duration is directly associated with increased MetS severity and indirectly linked to its reduction. Similarly, in women, SES has an inverse association, both direct and indirect, with MetS severity. Physical activity is directly and indirectly associated with decreasing MetS severity, while nap duration is directly related to increased severity and indirectly linked to its reduction.

The study identifies sleep duration as a mediating variable that not only directly impacts MetS severity but can also be influenced by age, SES, physical activity, obesity, night shift work, and nap duration. To prevent an increase in MetS severity, it is suggested to regulate sleep duration and consider controlling factors such as SES, physical activity, obesity, night shift work, and nap duration.

## Data Availability

All data generated and analyzed during this study are included in the manuscript.

## Abbreviations

MetS: Metabolic syndrome
SEM: structural equation model
RaNCD: Ravansar Non-communicable Disease
T2DM: Type 2 diabetes mellitus
LDL: Low-density lipoprotein
HDL: High-density lipoprotein
SES: Socio-economic status
PERSIAN: Prospective epidemiological research study in Iran
CVDs: Cardiovascular diseases
PCA: Principal component analysis
BMI: body mass index
BFM: Body Fat Mass
WHR: Waist hip ratio
WC: Waist circumference
CFI: comparative fit index
IFI: Incremental fit index
NFI: Normed fit index
RMSEA: Root mean square error of approximation
MLE: maximum likelihood estimation
